# Peptide hydrogel based sponge patch for wound infection treatment

**DOI:** 10.3389/fbioe.2022.1066306

**Published:** 2022-12-15

**Authors:** Lanxin Li, Yuan Zhou, Peizhe Li, Qi Xu, Kaiyan Li, Hai Hu, Wei Bing, Zhijun Zhang

**Affiliations:** ^1^ School of Chemistry and Life Science, Changchun University of Technology, Changchun, China; ^2^ Department of Pharmacy, Taihe Hospital, Hubei University of Medicine, Shiyan, China; ^3^ College of Pharmacy, Hubei University of Traditional Chinese Medicine, Wuhan, China; ^4^ Key Laboratory of Surface and Interface Science of Polymer Materials of Zhejiang Province, Department of Chemistry, Zhejiang Sci-Tech University, Hangzhou, China; ^5^ Shanghai Beautyart Biotechnology Co., Ltd., Shanghai, China

**Keywords:** peptide hydrogel, sponge patch, bacterial infection, wound healing, medical dressings

## Abstract

Dressing with the function of anti-wound infection and promoting skin repair plays an important role in medicine, beauty industry, etc. In terms of anti-wound infection, traditional dressings, such as gauze, have problems such as excessive bleeding in the process of contact or removal, and slow wound healing due to poor biological compatibility. The development of new functional and biocompatible dressings has essential application value in biomedical fields. In this study, a new type of dressing based on polypeptide functional sponge patch was constructed. The porous sponge patch is made of antimicrobial peptide and medical agarose through gel and freeze-drying technology. *In vitro* antibacterial experiments and small animal skin wound infection model experiments show that the porous sponge has excellent antibacterial and anti-skin infection activities, as well as the function of promoting wound healing.

## Introduction

Bacterial infection is a common phenomenon in the process of wound healing, which will trigger severe inflammatory reactions and further slow healing and impose a burden on patients. Yang et al. (2021a) Antibacterial dressings are often used in the treatment of wound infections in daily life. Homaeigohar and Boccaccini. (2020) Traditional antibacterial dressings have the advantages of low cost and easy batch preparation, Deng et al. (2020) but there are also problems such as poor air permeability, insufficient absorption capacity of tissue exudate and lack of promoting wound healing function. Li et al. (2021) In recent years, with the development of material science, more and more dressings fabrication strategies have been reported, among which antibacterial dressings based on hydrogels have attracted much attention due to their simple preparation and easy functionalization. Lin et al. (2021) Antibacterial hydrogels usually contain two main structural units, one of which is the skeleton matrix of hydrogel (e.g., calcium alginate, Zhang et al. (2021) agarose (Hou et al., 2020) and gelatin (Koshy et al., 2016)), and the other is the active groups or additives with antibacterial function [e.g., cationic polymers, Huang et al. (2016) antibiotics (Hoque et al., 2018) and antibacterial nanoparticles (Li et al., 2019)]. By adding special active substances in the preparation process, antibacterial hydrogels can also be given more functions, including promoting wound healing, hemostasis and so on. Mahlapuu et al. (2016) In addition, antibacterial hydrogels contain a large amount of water and present a soft gel state, which makes them have an excellent affinity to skin tissue that can reduce the pain of patients. Yuan et al. (2017) Due to these advantages, various antibacterial hydrogels have been reported in recent years. Chauhan and Singh. (2020) Undoubtedly, these studies have greatly enriched the variety of antibacterial dressings and provided more options for resisting wound infection. Nevertheless, hydrogel-based antibacterial dressings also have non-negligible drawbacks, including poor air permeability, high storage and transportation costs, etc. How to overcome the disadvantages while retaining the advantages of antibacterial hydrogels is the direction to construct new antibacterial dressings.

Sponge material is a kind of soft and light porous material. Yang et al. (2021b) The lightweight texture of sponge material makes it convenient for transportation and storage, and its porous characteristics make it have good air permeability and strong solution adsorption capacity, which are precisely what antibacterial hydrogels lack. Chao. (2019) The key to the preparation of sponge materials is the formation of material channels, which mainly include the template method, Zhao et al. (2021) foaming method, Li et al. (2018) porogen method, Asakura et al. (2017) phase separation method (Wang et al., 2018) and freeze-drying method. Cheng et al. (2022) Freeze-drying is a simple method to prepare sponge materials based on hydrogel. Chen et al. (2017) In this method, the internal water of hydrogel will be condensed into micro-ice crystals under low temperature to form porous structure. Then the freeze-drying technology is used to sublimate the ice crystals to obtain sponge materials. Using freeze-drying to construct antibacterial sponge based on antibacterial hydrogel can not only retain the function of antibacterial hydrogel, but also make it have the advantages of sponge permeability and solution adsorption capacity. Obviously, the hydrogel-based antibacterial sponge is an ideal antibacterial dressing. Unfortunately, seldom studies based on hydrogel antibacterial sponges have been reported up to now.

**SCHEME 1:**
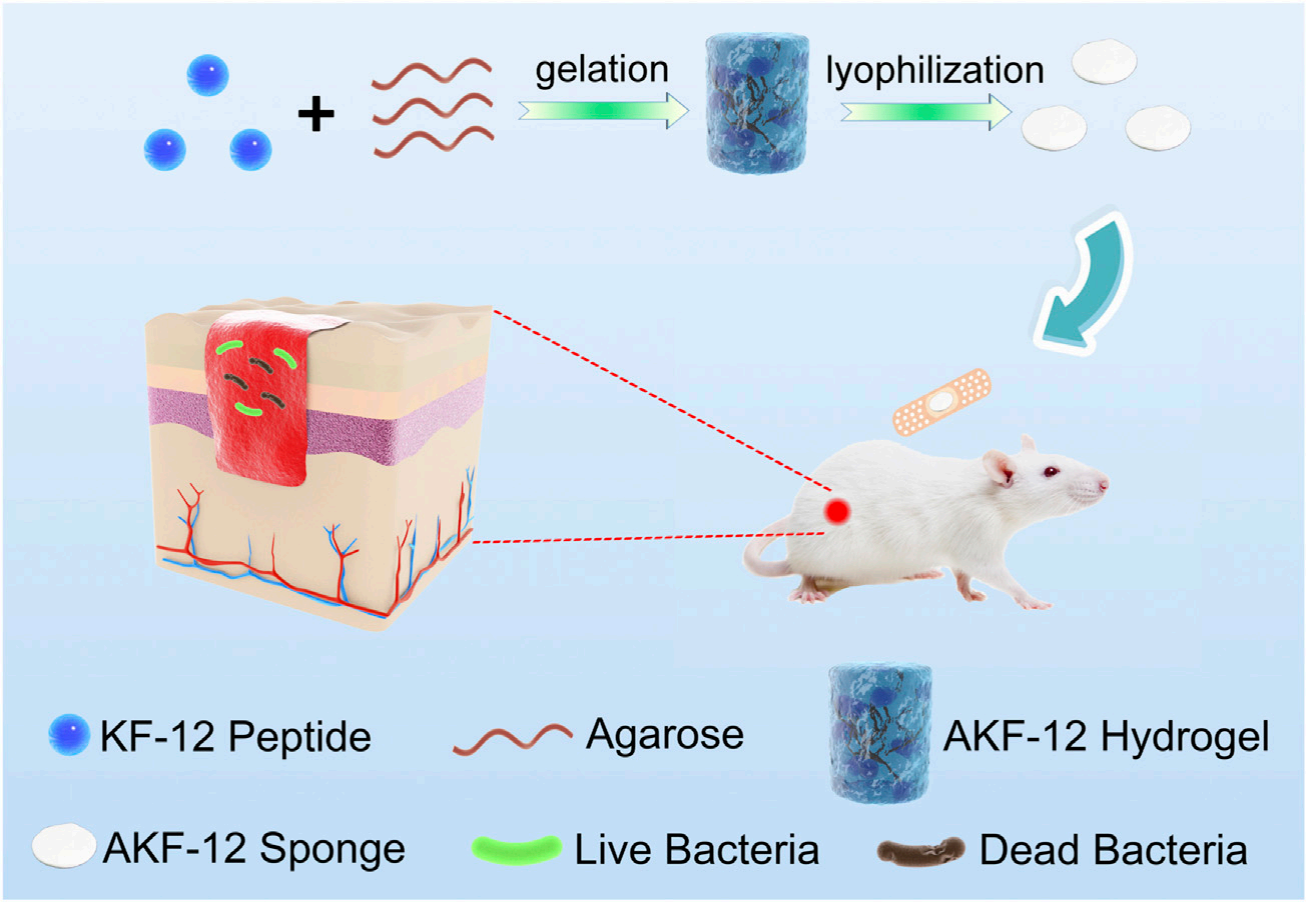
Schematic illustration of the preparation and antibacterial mechanism of the nanocomposite.

In this paper, we constructed the hydrogel (AKF-12H) with agarose as the skeleton matrix and antimicrobial peptide (KKLRLKIAFKFF, KF-12) as the antibacterial active ingredient. Subsequently, the AKF-12H was transformed into the antibacterial sponge (AKF-12S) by freeze-drying. And finally, the composition, morphology, porous properties, biocompatibility, antibacterial activity, anti-wound infection function and wound healing effect of the AKF-12S were systematically studied in Scheme 1.

## Materials and methods

### General information

2-Chlorotrityl Chloride Resin was purchased from GL Biochem (Shanghai) Ltd. Agarose was purchased from Sangon Biotech (Shanghai) Co., Ltd. Other reagents were purchased from Innochem. Fluorescence spectra were collected by an FL-970 fluorescence spectrometer (slit width 2.5 nm and PMT voltage 700 V). The group changes were analyzed using a Bruker Tensor 27 Fourier transform infrared spectrometer (FTIR). Scanning electron microscopy (SEM) was observed using a JSM-5610LV (JEOL). Fluorescence images were imaged by a NIB900-FL fluorescent microscope with a Nexcan-T6CCD digital camera (Nexcope, China). The colony counter icount 11 (Xun Shu, China) was used to count colony forming units. The purity of the peptides was monitored by HPLC with a UV 3100 detector (Dalian Elite Analytical Instruments Co., Ltd., China).

### Synthesis of peptides

Based on the standard Fmoc-based solid-phase peptide synthesis (SPPS) method, manual synthesis was carried out on 2-Chlorotrityl Chloride Resin (loading capacity: 1.082 mmol/g). Generally, 2-CTC resin was swelled with anhydrous DCM under N_2_ for 1 h. The Fmoc-protected first amino acid (8.0 equiv, based on the initial loading of the resin) was dissolved in DCM, followed by dropwise addition of DIPEA (6.0 equiv). The unreacted resin block was reacted with DIPEA in (methanol:DCM = 1:1) for 15 min. Then it was washed with DMF (5 times). Fmoc-groups were deprotected using piperidine (20% v/v in DMF). The remaining protected amino acids (3 equiv) were activated in anhydrous DMF for 2 h in the presence of HBTU (3 equiv) and DIPEA (6 equiv). Each amino acid eluted was washed with DMF (5 times). The peptide was cleaved from the resin with a mixture of TFA/H_2_O/TPS (95:2.5:2.5) for 40 min, the peptide solution was concentrated by rotary evaporation, then the crude peptide was precipitated in ice water with diethyl ether, centrifuged and retained. The purity of the obtained peptide was >85% as determined by RP-HPLC (CST Daiso C_18_ Column, 250 × 4.6 mm, 5 μm), MeOH (0.1% of TFA)/H_2_O (0.1% of TFA) was used as the mobile phase, and the detection was performed using ultraviolet light at 220 nm.

### Preparation of peptide hydrogel (AKF-12H) and sponge patch (AKF-12S)

First of all, agarose was dissolved in deionized water under a high temperature water bath. Then different amount of the antimicrobial peptide stock solution (25 mg/mL in methanol) was added to the dissolved agarose (around 40°C) in an EP tube. The final concentration of the agarose was fixed at 2% w/v, and the volume ratio of KF-12 stock solution was changed from 0%, 2%, 5%, 8%, 10%, 15%–20%, in which the final concentrations of the peptide were 0, 0.5, 1.25, 2, 2.5, 3.75, 5 mg/mL, respectively. The peptide hydrogel was obtained by cooling the mixture at 4°C for 1 h. Then the hydrogel was cut into slices and froze at −80°C for 4 h. Finally, the solidified hydrogel was lyophilized to form the sponge patch.

### Bacterial culture and antibacterial activity

*Staphylococcus aureus* (Gram-positive) and *Escherichia coli* (Gram-negative) were selected as model strains, respectively*.* Monocolonies of the bacteria on solid agar plates were transferred to 2 mL of LB medium and shaken under 150 rpm at 37°C for 12 h. Then, the bacterial solution was diluted to 0.5 at an optical density of 600 nm to evaluate its antibacterial activity. Agarose sponge (control) and AKF-12 sponge with different final concentrations of peptide (0.5, 1.25, 2, 2.5, 3.75, 5 mg/mL) were treated by spread plate method. Here, 3 and 30 min were selected for comparison. The sponge was placed in the bacterial solution with the same dilution multiple and mixed evenly, and 20 μL was transferred to the solid medium. After being placed in the incubator at 37°C for 12 h, the growth of microbial colonies was imaged and counted.

### Bacterial live/dead assay

The bacterial activity was determined by LIVE/DEAD™ BacLight™ bacterial viability kit (Thermo Fisher Scientific), which contains two dyes SYTO 9 and propidium iodide (PI). The agarose sponge group was used as the negative control, and the AKF-12 sponge (the volume ratio of KF-12 stock solution was 20% with final peptide concentration of 5 mg/mL) was then incubated with bacterial suspensions of *Escherichia coli* and *Staphylococcus aureus*, respectively. After incubation, each sample was treated with a mixture of SYTO 9 (Ex/Em = 480/500 nm) and propidium iodide (PI, Ex/Em = 490/635 nm) (2:1 v/v) for 20 min in the dark. After that, 10 μL of the stained samples were placed on a slide, which was inverted and photographed with fluorescence microscope. (100×, oil objective)

### Cytotoxicity assays

First, A549 cells were inoculated in a 96-well plate and incubated with sponge with different concentrations for 24 h. Then, 3-(4,5-dimethylthiazol-2-yl)-2,5-diphenyltetrazolium bromide (MTT) solution (5 mg/mL) was added after the replacement of fresh medium and incubated in an incubator at 37°C for 4 h. Finally, the supernatant was removed and 150 μl DMSO was added to each well to dissolve the purple formazan, then were shaken for 15 min. The absorbance was measured at 490 nm using a microplate reader. The agarose sponge group was used as the control group, and three parallel groups were set for each well.

### *In vivo* wound healing assay

All the animals have been approved by the Ethical Committee for Animal Experiments of Zhejiang Sci-Tech University, and all procedures have followed the guiding principles for animal experiments of Zhejiang Sci-Tech University. Female Kunming mice (6–8 weeks) were selected for this experiment. A mouse skin wound infection model was established. A wound with a diameter of about 6 mm was made on the back of each mouse and infected with 10 μl *E. coli* (10^6^ CFU/mL). After being infected, mice (6–8 weeks) were divided into four groups (three mice per group) with different treatments: *E. coli* only, ordinary band-aid, agarose sponge and sponge AKF-12S (KF-12 peptide concentration was 5 mg/mL) groups, respectively. Photographs were taken daily to record wound healing.

### H&E staining

All mice were sacrificed and the infected sites of the wound were collected after 10 days. The collected wound infection sites were fixed with 10% neutral buffered formalin, embedded in paraffin, sectioned and stained with hematoxylin and eosin (H&E). Finally, the tissue sections were observed by optical microscope.

## Results and discussion

### Synthesis and characterization of peptides

**FIGURE 1:**
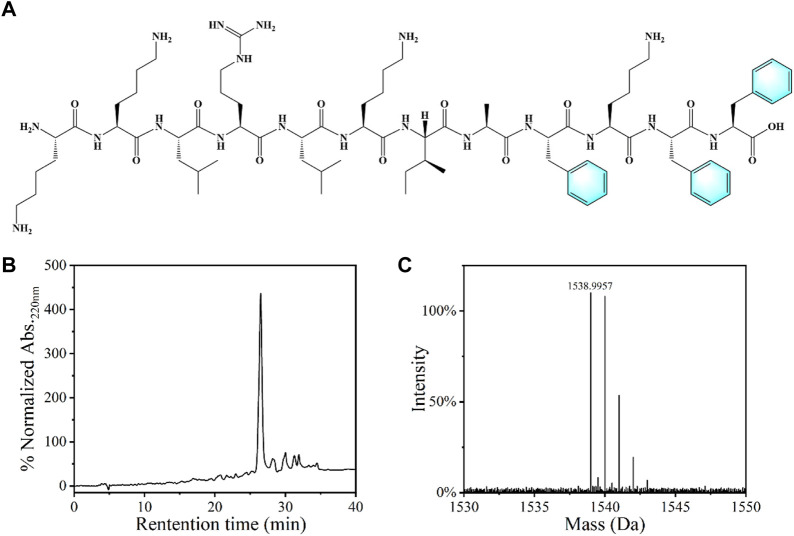
**(A)** Chemical structure of the KF-12 peptide: KKLRLKIAFKFF. **(B)** The HPLC spectra and **(C)** mass spectrogram of the KF-12 peptide.

Agarose is a natural polysaccharide with excellent biological safety, chemical stability and mechanical properties, which has been frequently used in biomedical field. Lee and Yun. (2018) Antimicrobial peptides (AMPs) are usually positively charged polypeptides with strong antibacterial properties and good biosafety, and not easy to generate bacterial resistance, which have been widely used in the development of new antibacterial materials. Brogden. (2005) In view of the advantages of agarose and AMPs, we choose them as the matrix and active substances for antibacterial hydrogel and antibacterial sponge fabrication. The dodecyl peptide with a specific sequence (KKLRLKIAFKFF, KF-12) was prepared by the solid phase peptide synthesis method and utilized as the antibacterial component, as shown in Figure 1A. HPLC and HRMS (High Resolution Mass Spectrometry) analysis indicate the purity of the obtained peptide was higher than 85% (Figures 1B,C).

### Prepare and characterization of peptide hydrogel (AKF-12H) and the sponge (AKF-12S)

**FIGURE 2:**
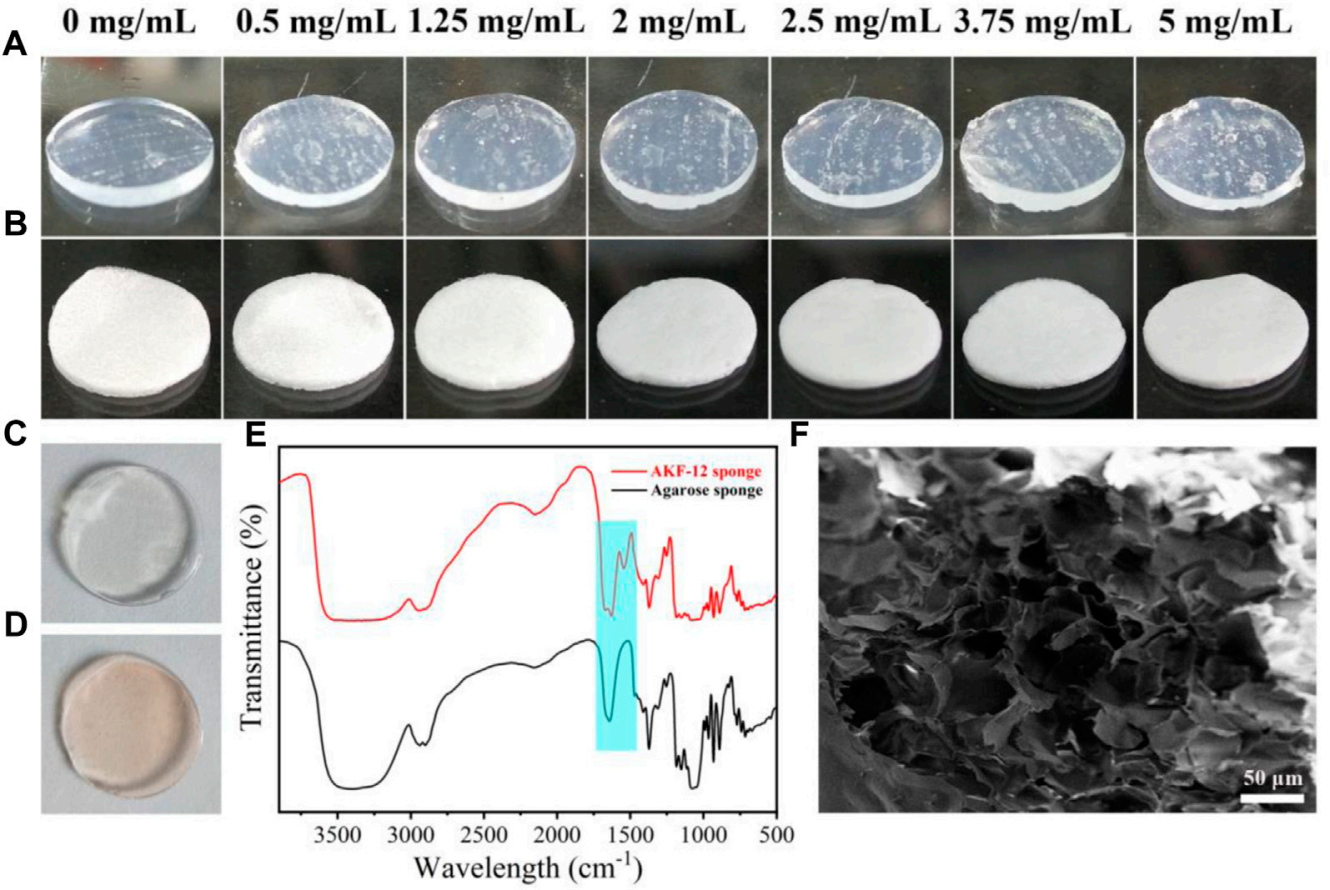
Images of peptide hydrogel AKF-12H prepared by different amounts of KF-12 **(A)** and the corresponding sponge AKF-12S **(B)**. FITC labeled agarose hydrogel **(C)** and AKF-12H hydrogel **(D)**. **(E)** FTIR spectrum of agarose sponge and sponge AKF-12S. **(F)** SEM images of sponge AKF-12S.

The peptide hydrogel (AKF-12H) was prepared by adding with different amounts of KF-12 into hot agarose aqueous solution and coagulating at low temperature. As shown in Figure 2A, the hydrogel of AKF-12H has a colorless and translucent appearance. The white sponge AKF-12S was obtained by freeze-drying the hydrogel (Figure 2B). FITC labeling results showed that only the peptide KF-12 doped hydrogel could be uniformly labeled with FITC (Figures 2C,D), indicating that KF-12 was uniformly distributed and stably bound in the composite without dissociation (Supplementary Figures S1A,B). The sponge is soft and light, and the components were determined by FTIR. As shown in Figure 2E, the broad peak at 3400 cm^−1^ is ascribed to the stretching vibration of -NH_2_ and -OH, the peaks at around the 1,545 cm^−1^ and 1,080 cm^−1^ are attributed to the skeleton vibration of benzene ring and the stretching vibration of C-O bond, and an obvious adsorption band of amide bond was observed at 1,675 cm^−1^. Cao et al. (2019) The FTIR spectra also clearly indicate the successful doping of the KF-12 peptide in the sponge. The porous structure of the sponge was further demonstrated by SEM imaging. A porous structure was clearly observed in the SEM image. The pore size of the sponge is tens of microns and is formed by the stacking of sheet structures (Figure 2F). The porous structure will facilitate the absorption of tissue fluid during the antibacterial process. Subsequently, the adsorption capacity of the sponge on liquid was verified through water absorption test. (Supplementary Figure S2) All of the sponges showed high water absorption ability, and the water absorption capacity of AKF-12S sponge is more than 20 times of its own mass and has more than 90% porosity. (Supplementary Figure S3) Finally, the mechanical properties of the hydrogel and sponge was investigated. As shown in the stress-strain curves (Supplementary Figure S4), when AKF-12H hydrogel is compressed to 20% strain, the sample is damaged and has poor compressive resistance. On the contrary, the AKF-12S sponge can be compressed to 98% strain and will not be damaged, showing excellent compressive resistance.

### Antibacterial activities

**FIGURE 3:**
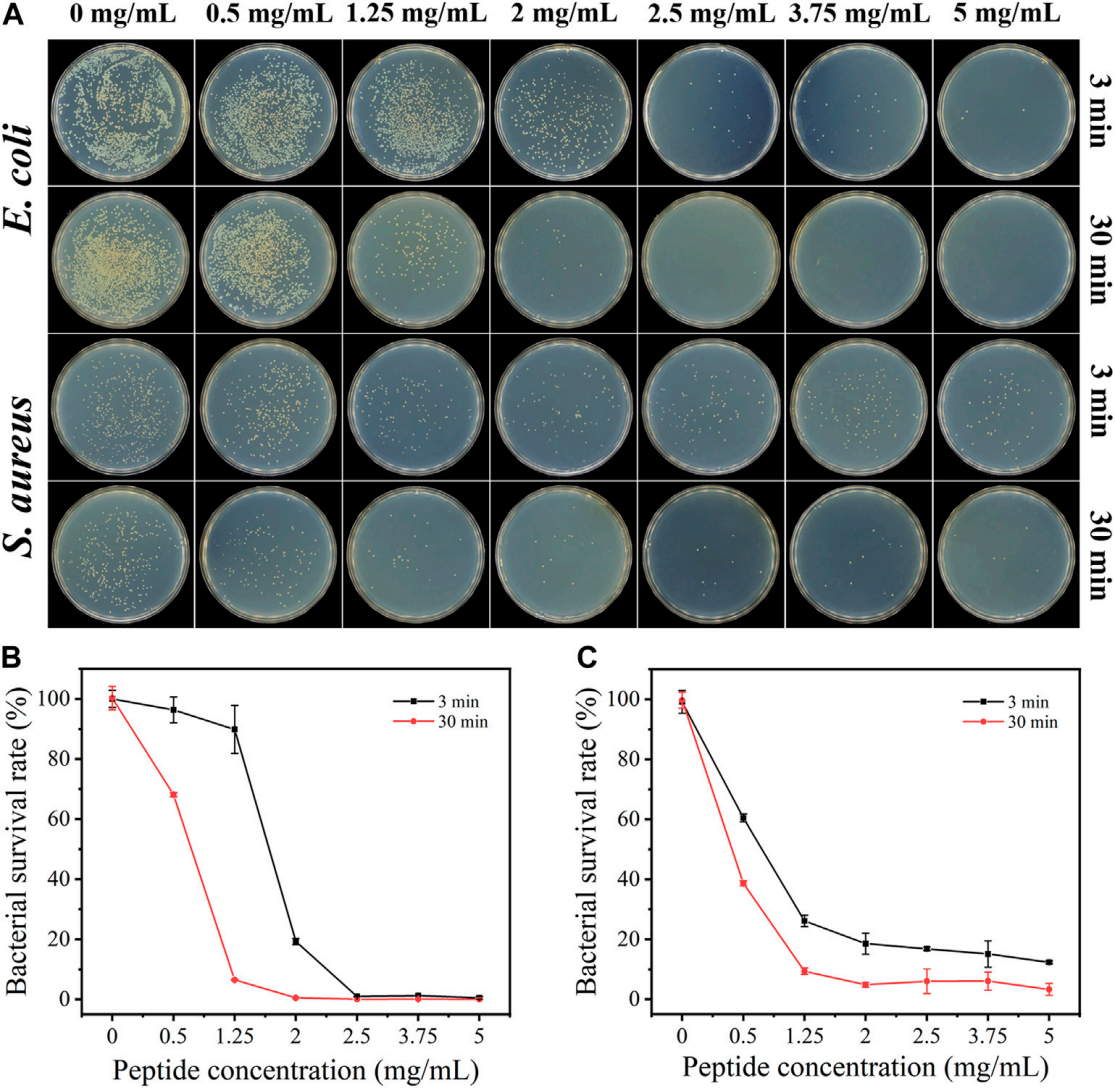
**(A)** Colony images of *E. coli* and *S. aureus* after treated with AKF-12S containing different concentrations of antimicrobial peptide at 3 and 30 min. Bacterial survival of *E. coli* **(B)** and *S. aureus* **(C)** after incubated with different concentrations of antimicrobial peptide.

The antibacterial activity of the KF-12 peptide was first investigated. As shown in Supplementary Figure S5, the minimum inhibitory concentration (MIC) values against *S. aureus* and *E. coli* were measured to be 128 μg/mL (over 80% inhibition) and 64 μg/mL (100% inhibition), respectively. The antibacterial activities of the sponge AKF-12S with different antimicrobial peptide concentrations against *E. coli* and *S. aureus* were then studied by the plate counting method. Briefly, the bacterial solution was dropped into the sponge, after incubated for a certain time the sponge with bacteria was crushed and diluted. Then the dispersion was transferred to LB agar plates, incubated for 12 h at 37°C and the colonies were counted. The antibacterial effect was assessed by the number of colonies forming units on LB agar plates. As shown in Figure 3, for *E. coli*, the increase of antimicrobial peptide concentration in sponge AKF-12S led to a significant reduction in bacterial survival rate. With the incubation time extended to 30 min, the sponge AKF-12S with 1.25 mg/mL of antimicrobial peptide could effectively kill more than 93% of bacteria (Figure 3B), which was more significant than that of 3 min. Surprisingly, almost no bacteria survived when the concentration of antimicrobial peptide in sponge AKF-12S increased to 2 mg/mL. A similar phenomenon was observed in *S. aureus*, which killed nearly 97% of bacteria with 30 min incubation at the antimicrobial peptide concentration of 5 mg/mL (Figure 3C). In contrast, bacterial survival in the control group remained stable. The above results indicated that the survival rate of bacteria decreased with the increase of the concentration of antimicrobial peptide in sponge AKF-12S and the prolongation of incubation time. In summary, the sponge AKF-12S has good antibacterial activity on both Gram-negative bacteria and Gram-positive bacteria.

### Bacterial live/dead assay

**FIGURE 4:**
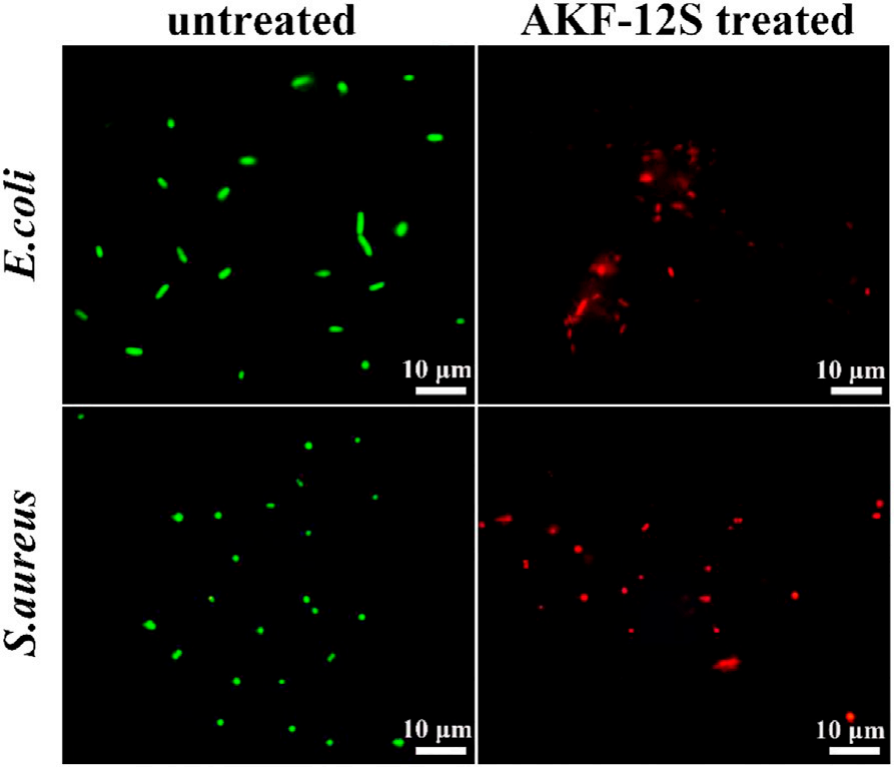
Live/dead staining of the untreated and sponge AKF-12S treated bacterial cells.

The antibacterial mechanism was initially discussed by live/dead staining with a LIVE/DEAD BacLight™ bacterial viability kit that contains SYTO 9 and PI. SYTO 9 is a green fluorescent nucleic acid dye that can permeate the membrane of live cells. PI penetrates and reddens the damaged cell membrane of late apoptotic cells and dead cells. As shown in Figure 4, untreated bacteria showed strong green fluorescence, while the sponge AKF-12S treated bacteria showed strong red fluorescence, indicating the significant damage of the bacterial membrane by the sponge AKF-12S and the destruction of bacterial membrane could be the reason for the activity of the sponge.

### Evaluation of antibacterial activity in skin wound infection

**FIGURE 5:**
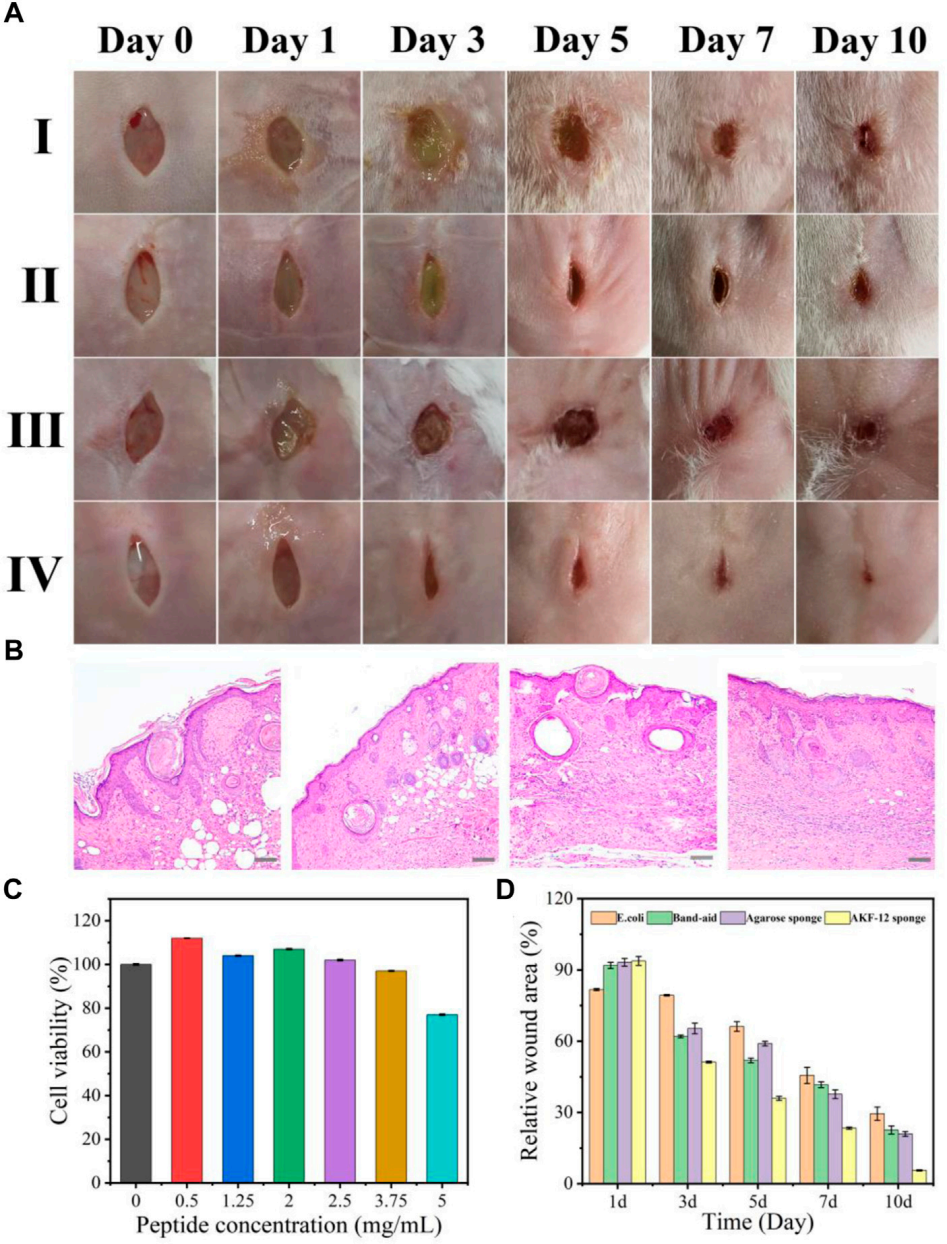
**(A)** Time-dependent images of *E. coli*-infected wounds on mice backs with different treatments. (Ⅰ: *E. coli* onl*y*, Ⅱ: ordinary band-aid, Ⅲ: agarose sponge, Ⅳ: sponge AKF-12S). **(B)** Histological studies with H&E staining of skin tissues harvested from mice after 10 days of treatment. (from left to right: *E. coli only*, and *E. coli* treated with ordinary band-aid, agarose sponge, sponge AKF-12S). Scale bars represent 100 µm. **(C)** Viability of A549 cells in the presence of the sponge AKF-12Ss. **(D)** Relative wound area of different groups after 10 days of treatment (Measured by ImageJ software analysis).

Considering the high requirements of wound application on the safety of dressings, we further investigated the cytotoxicity of the sponge AKF-12S on mammalian cells. The cell viability of A549 cells exposed to sponge AKF-12S was determined by MTT assay. As shown in Figure 5C, the cell viability can still remain above 80% even after incubation with sponge AKF-12S of the highest antimicrobial peptides concentration, which fully proves that the sponge AKF-12S has good biocompatibility. After that, we constructed a mouse skin wound infection model to further verify the practical performance of the antibacterial sponge patch. The wound size and recovery degree were used to evaluate the ability of the sponge AKF-12S to promote wound healing. As shown in Figures 5A,D, the wound area of the sponge AKF-12S group decreased to 50% on the third day, while the other groups had some yellow pus outflow accompanied by some redness. On the 10th day, eschars had been formed on the wounds of the sponge AKF-12S group, and the healing speed was significantly faster than that of other groups (wound closure ratio was 96.31%), which was consistent with the *in vitro* antibacterial results.

Furthermore, we collected the wound tissue on the 10th day and obtained its optical micrograph after H&E staining and analyzed the healing state. As shown in Figure 5B, for the sponge AKF-12S group, there were fewer inflammatory cells, more compact collagen fibers and elongated epithelial cells, indicating that the re-epithelization of the wound part was enhanced, which was a crucial part of the healing process. However, inflammation and epidermal hyperplasia were observed in other groups. All above results suggested the sponge AKF-12S has multifunctional characteristics to effectively resist bacterial infection and reduce inflammatory reactions in the process of wound healing, so as to promote wound healing.

## Conclusion

In conclusion, antimicrobial peptides were successfully synthesized, and stable hydrogels were prepared using peptides and agarose as raw materials. Then, the porous sponge was obtained by freeze-drying. The porous sponge showed good antibacterial properties against both Gram-negative and Gram-positive bacteria. In addition, the porous sponge, as a wound patch, can effectively prevent skin wound infection in mice and has an obvious effect of promoting wound healing. This study not only shows a multifunctional peptide sponge patch with antibacterial and wound healing effects, but also provides a new idea for the construction of polypeptide hydrogels and sponge materials.

## Data Availability

The original contributions presented in the study are included in the article/Supplementary Material, further inquiries can be directed to the corresponding authors.
