# Supplementary material for: Peptide hydrogel based sponge patch for wound infection treatment
Source: Front Bioeng Biotechnol. 2022 Dec 15;10:1066306. doi: 10.3389/fbioe.2022.1066306 (PMC9797970; doi:10.3389/fbioe.2022.1066306)
Supplement: Supplementary file 1 [file DataSheet1.PDF]

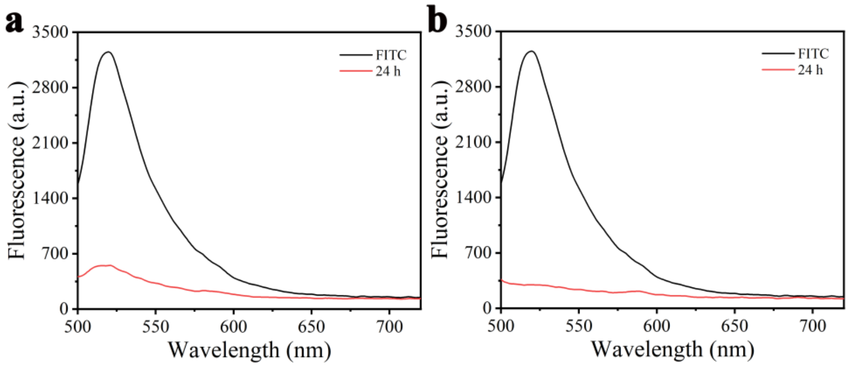

Fig. S1. The fluorescence emission spectra of 0.01 μM FITC, and the aqueous solution soaked with AKF-12H (a) and AKF-12S (b) for 24 hours, respectively.

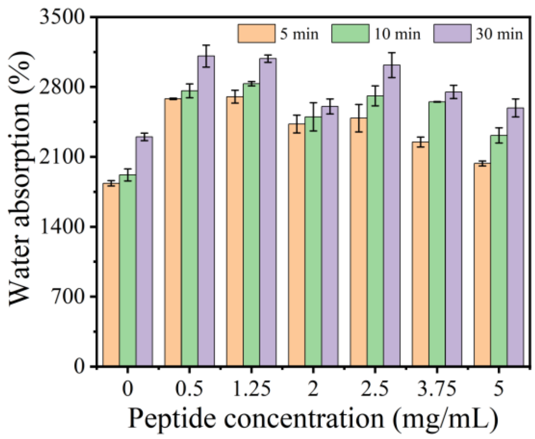

Fig. S2. The water absorption of the sponge AKF-12S.

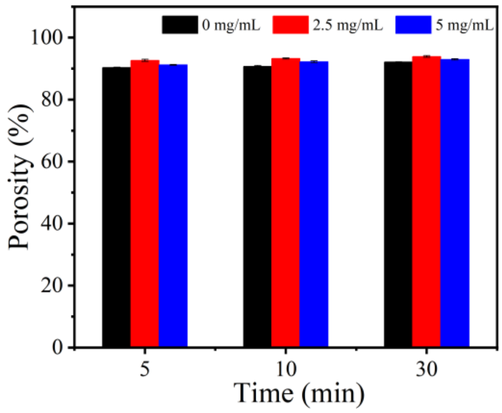

Fig. S3. The porosity of AKF-12S sponge.

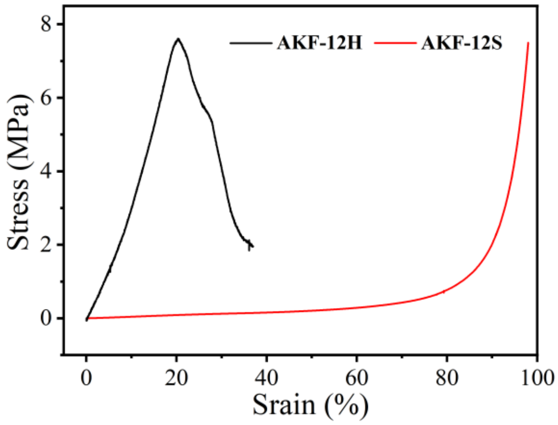

Fig. S4. The stress-strain curves of AKF-12H and AKF-12S.

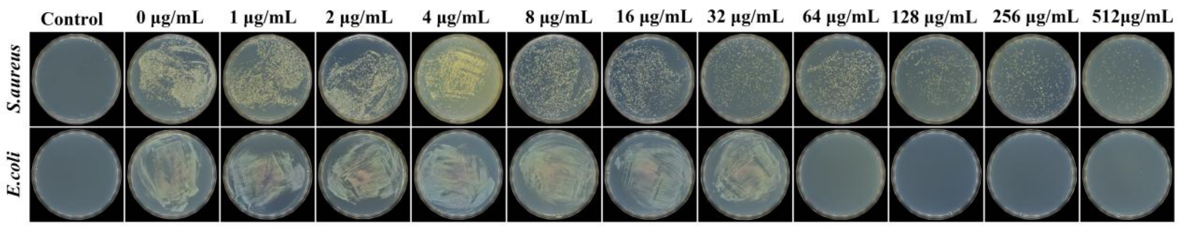

Fig. S5. Photographic images of the colonies of *E. coli* and *S.aureus* treated with different concentrations of KF-12 peptide at 37 °C for 18 h.
